# Vitamin D, Parathyroid Hormone and Their Associations with Hypertension in a Chinese Population

**DOI:** 10.1371/journal.pone.0043344

**Published:** 2012-08-16

**Authors:** Lihua Li, Xueyan Yin, Chaoyong Yao, Xuechuang Zhu, Xinhua Wu

**Affiliations:** Department of Internal Medicine, Dali University School of Medicine, Dali, Yunnan Province, China; Brigham and Women’s Hospital and Harvard Medical School, United States of America

## Abstract

**Background:**

Conflicting reports support or refute an association between vitamin D deficiency with high levels of parathyroid hormone (PTH) and raised blood pressure or hypertension.

**Objective:**

To explore the associations of serum vitamin D and PTH levels with blood pressure and risk of hypertension in a Chinese population.

**Methods:**

A population-based cross-sectional study was conducted among 1,420 Chinese participants, aged 20–83 years, in 2010. Anthropometric phenotypes and blood pressure were evaluated. Serum lipids, 25-hydroxyvitamin D [25(OH)D] and PTH were measured.

**Results:**

One thousand four hundred and twenty participants, including 566 women (39.9%), were evaluated in 2010. Four hundred and eighty seven were hypertensive (34.3%), of whom 214 (43.9%) received antihypertensive treatment. The median concentrations of serum 25(OH)D and PTH were 22.0 ng/ml and 2.83 pmol/l, respectively. Serum 25(OH)D and natural log of PTH levels were not independently associated with blood pressure in a multivariable adjusted linear regression analysis of 1,206 participants not receiving antihypertensive treatment (*P*>0.05). In logistic regression analyses, serum 25(OH)D levels were not associated with risk of hypertension in single and multiple regression models. One unit increments of natural log of PTH levels were significantly associated with risk of hypertension in the crude model (OR = 1.78, 95% confidence interval 1.38–2.28, *P*<0.0001) and model adjusted for age and sex (OR = 1.41, 95% confidence interval 1.08–1.83, *P* = 0.01). However, these associations were attenuated and became nonsignificant (OR = 1.29, 95% confidence interval 0.98–1.70, *P* = 0.07) after further adjustment for body mass index, current alcohol intake, current smoking, glomerular filtration rate and family history of hypertension.

**Conclusions:**

Serum vitamin D and PTH levels are not independently associated with blood pressure or risk of hypertension in a Chinese population.

## Introduction

Hypertension is one of the important risk factors for cardiovascular disease, which is the major cause of morbidity and mortality worldwide. Vitamin D is known to regulate calcium and phosphate metabolism [1]. However, accumulating evidence suggests that vitamin D level is inversely related to blood pressure and risk of hypertension in observational studies in the Western populations [2], [3], [4], [5], [6], [7]. Evidence from the Chinese population is limited [8], [9], [10]. Because of racial and geographic differences in vitamin D status [11], and the fact that Chinese have not yet been exposed to food fortified with vitamins and seldom use vitamin D supplements, it is not clear whether these findings from the Western populations can be extrapolated to Chinese individuals. In addition, some studies showing a significant association between vitamin D and risk of hypertension fail to adjust for serum parathyroid hormone (PTH) [2], [5], [12], an important calcium-regulating hormone. Some studies have indicated that serum PTH was positively correlated with blood pressure and the risk of hypertension [4], [13], even in the normal PTH range [14].

China, with a rapidly developing economy and large aging population, is experiencing a growing pandemic of hypertension [15]. Vitamin D deficiency is common in middle-aged and elderly Chinese [8], [10]. However, little is known about the role of vitamin D deficiency in hypertension. Furthermore, no epidemiological study of hypertension has examined both serum vitamin D and PTH levels together in Chinese.

We hypothesized that lower levels of vitamin D and higher levels of PTH are associated with blood pressure and the risk of hypertension in Chinese. 25(OH)D is the main storage form of vitamin D in the body and the most commonly used marker to evaluate vitamin D status. We conducted this cross-sectional study to evaluate the associations of serum 25(OH)D and serum PTH with blood pressure and the risk of hypertension in a Chinese population.

## Methods

### Ethics Statement

The Ethics Committee of the Affiliated Hospital of Dali University approved the study protocol. All subjects gave written informed consent. The study complied with the guidelines in the Declaration of Helsinki.

### Study Population

The present analysis was based on a cross-sectional study in the framework of comprehensive cardiovascular health examinations for all employees of a factory in Dali (25°N) of Yunnan Province, a small city in southwest of China. We invited all employees and retired workers to take part in this study from March to May 2010. Of the 1,643 eligible individuals, 1,443 (87.8%) agreed to participate in the study. In addition, 23 participants were excluded for inadequate blood sample to test 25(OH)D or PTH (n = 8), or lack of demographic data (n = 15). Thus, a total of 1,420 participants was included in the present analysis.

**Table 1:** Characteristics of participants without antihypertensive treatment according to quartiles of serum 25(OH)D levels.

	25(OH)D (ng/ml)				
	<16.8	16.8–22.5	22.6–28.3	>28.3	
	(n = 295)	(n = 310)	(n = 301)	(n = 300)	*P*
25(OH)D, ng/ml	12.8±2.8	19.7±1.6	25.3±1.6	34.0±4.8	–
PTH, pmol/l	3.12	2.89	2.54	2.52	0.0001
	(2.04–4.42)	(1.91–3.99)	(1.80–3.61)	(1.65–3.66)	
Study outcome					
Systolic BP, mmHg	124.4±18.6	124.7±19.4	124.6±17.9	123.4±17.5	0.84
Diastolic BP, mmHg	77.7±11.6	78.4±11.1	78.8±11.5	77.7±11.0	0.60
Pulse pressure, mmHg	46.6±11.5	46.3±12.3	45.8±10.4	45.7±10.5	0.69
Possible confounders					
Male, %	50.2	58.1	65.5	67.7	<0.0001
Age, years	49.5±13.4	47.0±11.9	45.9±11.9	45.6±12.3	0.0004
BMI, kg/m^2^	23.0±3.2	23.4±3.4	23.3±3.2	23.1±3.2	0.42
Total cholesterol, mmol/l	5.6±1.2	5.4±1.1	5.4±1.0	5.3±1.0	0.02
Serum glucose, mmol/l	5.4±2.0	5.4±1.8	5.2±1.7	5.2±1.5	0.23
GFR, ml/min.1.73 m^2^	97.6±19.3	95.3±18.4	97.2±21.0	94.2±18.2	0.10
Current alcohol intake, %	16.3	21.6	31.2	25.3	0.0002
Current smoking, %	31.2	34.8	40.5	41.0	0.04
Hypertension, %	23.4	22.3	22.6	22.3	0.99

Data are mean ± standard deviation, or median (interquartile range), or percentages. BMI, body mass index; BP, blood pressure; GFR, glomerular filtration rate.

### Data Collecting

One physician measured each participant’s blood pressure three times consecutively using an automatic blood pressure monitor (Omron HEM 7011), after the subjects had rested for at least 5 minutes in the sitting position. The three blood pressure readings were averaged for analysis. The same observer also administered a questionnaire to collect information on medical history, smoking habits, alcohol consumption and the use of medications. A trained technician performed anthropometric measurements, including body height, body weight, and waist and hip circumference. Hypertension was defined as a sitting blood pressure of at least 140 mmHg systolic or 90 mmHg diastolic, or as the use of antihypertensive drugs. Current smoking was defined as at least 1 cigarette smoked per day. Current alcohol intake was defined as consuming at least 1 drink per week. Body mass index was defined as a ratio of the body weight in kilograms to the square of the height in meters. Glomerular filtration rate (GFR) was calculated using the Modification of Diet in Renal Disease (MDRD) Study equation as: GFR = 186× (serum creatinine, mg/dl)^− 1^ ^.154^× (age, years)^−0^ ^.203^× (0.742 if female) [16].

### Laboratory Methods

Venous blood samples were drawn after overnight fasting for the measurement of serum glucose, creatinine and total cholesterol. Serum was also stored at −30°C for measurement of 25(OH)D and PTH. Serum 25(OH)D was determined by radioimmunoassay (Diasorin 25-hydroxyvitamin D ^125^I RIA Kit, Stillwater, Minnesota, USA) in the Clinical Laboratory of the Affiliated Hospital of Dali University. The intra- and interassay coefficients of variance were 6.0% and 5.6%. Serum PTH assay was performed in the same laboratory by the chemiluminescence method (DPC 2000, Siemens, Germany). The intra- and interassay coefficients of variance were 3.9% and 6.0%.

**Table 2:** Characteristics of participants without antihypertensive treatment according to quartiles of natural log of PTH levels.

	Natural log of PTH				
	<0.62	0.62–1.01	1.02–1.36	>1.36	
	(n = 303)	(n = 302)	(n = 299)	(n = 302)	*P*
25(OH)D, ng/ml	24.4±8.1	23.3±8.1	22.5±8.3	21.6±8.2	0.0002
PTH, pmol/l	1.41	2.29	3.27	4.98	–
	(1.11–1.63)	(2.05–2.50)	(3.00–3.52)	(4.35–6.09)	
Study outcomes					
Systolic BP, mmHg	121.2±15.6	122.3±17.3	125.6±19.0	128.0±20.6	<0.0001
Diastolic BP, mmHg	76.7±9.8	77.3±10.8	78.4±11.0	80.2±13.0	0.0006
Pulse pressure, mmHg	44.5±10.0	44.9±10.1	47.2±12.2	47.8±12.1	0.0003
Possible confounders					
Male, %	57.4	57.0	63.6	63.6	0.16
Age, years	43.3±12.4	45.6±11.4	48.7±13.0	50.4±12.0	<0.0001
BMI, kg/m^2^	22.9±3.1	23.0±3.1	23.5±3.5	23.6±3.3	0.01
Total cholesterol, mmol/l	5.4±1.1	5.4±1.1	5.4±1.0	5.5±1.1	0.85
Serum glucose, mmol/l	5.2±1.7	5.2±1.6	5.3±1.3	5.6±2.3	0.03
GFR, ml/min.1.73 m^2^	97.2±18.1	96.7±18.3	95.8±19.5	94.6±21.0	0.37
Current alcohol intake, %	22.4	23.8	22.7	25.5	0.81
Current smoking, %	33.7	37.8	38.1	38.1	0.61
Hypertension, %	15.2	19.5	25.8	30.1	<0.0001

Data are mean ± standard deviation, or median (interquartile range), or percentages. BMI, body mass index; BP, blood pressure; GFR, glomerular filtration rate.

### Statistical Methods

For database management and statistical analysis, we used SAS software (version 9.1.3, SAS Institute, Cary, NC). Because the distribution of serum PTH levels was skewed, PTH values were logarithmically transformed. Quantitative variables were summarized as mean and SD or median and interquartile range if the distribution of the variable was skewed. The differences between means were tested using the one-way analysis of variance (ANOVA). The differences between proportions were tested using the chi-square test. We restricted linear and logistic regression analyses to participants not receiving antihypertensive treatment to remove the influence of antihypertensive treatment on blood pressure. We performed unadjusted linear and logistic analyses in single regression and adjusted analyses in multiple regression after adjustment for age and sex (in all participants), full adjustment for body mass index, current alcohol intake (yes/no), current smoking (yes/no), glomerular filtration rate and family history of hypertension. The interactions between serum 25(OH)D, natural log of PTH and other factors were examined by adding an interaction term to the linear regression analysis. *P* values were two-sided and *P*≤0.05 was considered significant.

## Results

One thousand four hundred and twenty participants included 566 women (39.9%). Four hundred and eighty seven participants were hypertensive (34.3%), of whom 214 (43.9%) received antihypertensive treatment. The prevalence of hypertension was 37.6% and 29.3% for men and women, respectively. The median concentrations of serum 25(OH)D and PTH for the entire group were 22.0 ng/ml and 2.83 pmol/l, respectively.

Characteristics of the 1,206 participants without antihypertensive treatment are shown in Table 1 and Table 2 according to the quartiles of serum 25(OH)D and natural log of PTH levels. Compared with participants with higher serum 25(OH)D levels, those with lower 25(OH)D levels had higher serum PTH levels, were older, were fewer men, and less commonly had current alcohol intake and current smoking (*P*≤0.04). Compared with participants with lower natural log of PTH levels, those with higher natural log of PTH levels had lower serum 25(OH)D levels, higher systolic and diastolic blood pressure, higher pulse pressure, higher serum glucose and higher prevalence of hypertension, a greater body mass index and were older (*P*≤0.03).

Serum 25(OH)D levels were not statistically associated with either systolic or diastolic blood pressure (*P*>0.05) in linear regression analyses in the 1,206 participants without antihypertensive treatment both in unadjusted and adjusted models (*P*≥0.10, Table 3). In contrast, natural log of PTH levels were positively associated with systolic and diastolic blood pressure in a crude model and in the model adjusted for age and sex. However, these associations were attenuated significantly and became nonsignificant when further adjusted for current alcohol intake, current smoking, GFR and family history of hypertension (Table 4). To examine the consistency of these associations, we performed subgroup analyses stratified by gender, and the results were consistent (Table 3, Table 4). There were no significant interactions of serum 25(OH)D and natural log of PTH levels with age, sex, body mass index, GFR, current alcohol intake, current smoking and family history of hypertension in relation to systolic and diastolic blood pressure (all *P* interactions >0.05).

**Table 3:** Associations of serum 25(OH)D levels with blood pressure in participants without antihypertensive treatment.

	All (n = 1206)			Men (n = 728)			Women (n = 478)		
	RC	SE	*P*	RC	SE	*P*	RC	SE	*P*
Systolic BP, mmHg									
Model 1	−0.0626	0.0640	0.32	−0.0948	0.0792	0.23	−0.1739	0.1048	0.10
Model 2	−0.0226	0.0591	0.70	−0.0118	0.0763	0.88	−0.0448	0.0919	0.63
Model 3	−0.0089	0.0563	0.87	−0.0145	0.0732	0.84	0.0042	0.0883	0.96
Diastolic BP, mmHg									
Model 1	−0.0107	0.0394	0.79	−0.0612	0.0493	0.21	−0.0409	0.0621	0.51
Model 2	−0.0280	0.0385	0.47	−0.0425	0.0495	0.39	−0.0048	0.0609	0.94
Model 3	−0.0158	0.0359	0.66	−0.0444	0.0459	0.33	0.0305	0.0585	0.60

Model 1: crude model. Model 2: model 1 adjusted for age and sex (in all participants).

Model 3: model 2 further adjusted for body mass index, current alcohol intake (yes/no), current smoking (yes/no), glomerular filtration rate and family history of hypertension. RC: regression coefficient; SE: standard error; BP: blood pressure.

**Table 4:** Associations of serum natural log of PTH levels with blood pressure in participants without antihypertensive treatment.

	All (n = 1206)			Men (n = 728)			Women (n = 478)		
	RC	SE	*P*	RC	SE	*P*	RC	SE	*P*
Systolic BP, mmHg									
Model 1	4.8429	0.9189	<0.0001	3.8059	1.3516	0.001	5.4959	1.4107	0.0001
Model 2	2.0527	0.8629	0.02	1.5751	1.1610	0.18	2.9102	1.2561	0.02
Model 3	1.0546	0.8222	0.20	0.4806	1.1146	0.67	1.9816	1.2026	0.10
Diastolic BP, mmHg									
Model 1	2.3082	0.5673	<0.0001	2.1579	0.7326	0.03	1.9319	0.8423	0.02
Model 2	1.4792	0.5610	0.01	1.7140	0.7521	0.02	1.2229	0.8347	0.14
Model 3	0.7392	0.5427	0.16	0.8392	0.6982	0.23	0.6491	0.7989	0.42

Model 1: crude model. Model 2: model 1 adjusted for age and sex (in all participants).

Model 3: model 2 further adjusted for body mass index, alcohol intake (yes/no), current smoking (yes/no), glomerular filtration rate and family history of hypertension. RC: regression coefficient; SE: standard error; BP: blood pressure.

Table 5 shows associations of serum 25(OH)D and natural log of PTH levels with the risk of hypertension in the 1,206 participants without antihypertensive treatment in logistic regression models. Serum 25(OH)D levels were not associated with the risk of hypertension in the crude model or adjusted models (*P*≥0.31). A one unit increment of natural log of PTH was significantly associated with a 1.78-fold increased risk of hypertension [OR = 1.78, 95% confidence interval (CI) 1.38–2.28, *P*<0.0001] in the crude model and a 1.41-fold increased risk of hypertension (OR = 1.41, 95%CI 1.08–1.83, *P* = 0.01) in model adjusted for age and sex, respectively. However, when body mass index, GFR, current alcohol intake, current smoking and family history of hypertension were further adjusted, these associations were attenuated and became nonsignificant (OR = 1.29, 95%CI 0.98–1.70, *P* = 0.07). Subgroup analyses stratified by gender yielded similar results.

**Table 5:** Associations (ORs with 95%CI) of serum 25(OH)D and natural log of PTH levels with the risk of hypertension in participants without antihypertensive treatment.

	All (n = 1206)	Men (n = 728)		Women (n = 478)		
	OR (95%CI)	*P*	OR (95%CI)	*P*	OR (95%CI)	*P*
25(OH)D, ng/ml						
Model 1	1.00 (0.98–1.01)	0.58	0.99 (0.97–1.01)	0.31	0.99 (0.96–1.02)	0.61
Model 2	1.00 (0.98–1.02)	0.87	1.00 (0.98–1.02)	0.98	1.01 (0.97–1.04)	0.76
Model 3	1.00 (0.98–1.02)	0.78	1.00 (0.98–1.02)	0.95	1.00 (0.97–1.04)	0.82
Natural log of PTH, pmol/l						
Model 1	1.78 (1.38–2.28)	<0.0001	1.72 (1.27–2.34)	0.0005	1.80 (1.16–2.79)	0.009
Model 2	1.41 (1.08–1.83)	0.01	1.38 (1.00–1.90)	0.05	1.50 (0.93–2.40)	0.09
Model 3	1.29 (0.98–1.70)	0.07	1.24 (0.89–1.75)	0.21	1.45 (0.89–2.35)	0.13

Model 1: crude model. Model 2: model 1 adjusted for age and sex (in all participants).

Model 3: model 2 further adjusted for body mass index, current alcohol intake (yes/no), current smoking (yes/no), glomerular filtration rate and family history of hypertension.

## Discussion

The main findings of our cross-sectional study are that serum 25(OH)D and PTH levels are not independently associated with blood pressure level or risk of hypertension in a Chinese population.

Our findings that serum 25(OH)D was not significantly associated with blood pressure level or risk of hypertension are consistent with some studies [17], [18], [19], [20], but not others [8], [10], [13]. In the Longitudinal Ageing Study Amsterdam, serum 25(OH)D was not associated with systolic blood pressure or diastolic blood pressure, or prevalence of hypertension in 1,205 men and women aged 65 years and older after adjustment for potential confounders [17]. In the Ranch Bernardo Study, serum 25(OH)D was not associated with prevalence of hypertension in both sexes [18]. Margolis *et al.* [19] reported 4,863 postmenopausal women recruited into the Women’s Health Initiative between 1993 and 1998. Over a 7 year period, serum levels of 25(OH)D did not correlated with changes in blood pressure, and evidence for an association with lower risk of incident hypertension was weak. Furthermore, a systematic review and meta-analysis of randomized trials was unable to demonstrate a statistically significant reduction in systolic or diastolic blood pressure associated with vitamin D [20].

Our finding is in contrast to three Chinese studies. Lu *et al*. [8] investigated associations of plasma 25(OH)D concentrations with metabolic syndrome among 3,262 middle-aged and elderly Chinese in Beijing (39°N) and Shanghai (31°N). In their study, mean concentration of 25(OH)D was 40.4 nmol/l (16.6 ng/ml), and 25(OH)D concentration was inversely related to diastolic blood pressure. In another study, 939 Chinese older men in Hong Kong (22°N) had mean serum 25(OH)D levels and median serum PTH levels of 77.9 nmol/l (31.2 ng/ml) and 4.1 pmol/l, respectively [9]. Chan *et al.* [9] found a significant association between serum PTH and blood pressure, but not serum 25(OH)D. Furthermore, in the Shanghai Women’s and Men’s study, median 25(OH)D level was 34.7 nmol/l (13.9 ng/ml). 25(OH)D levels were inversely related to individual blood pressure parameters and hypertension among middle-aged and elderly men, but not among women [10]. The inconsistency among these four Chinese studies may be partially due to different study populations, geographic, and seasonal differences. Our study was conducted in relatively younger participants (mean age 49.1 years) in Dali (25°N) in summer. Serum 25(OH)D was significantly higher than those in two other studies, but lower than those in Hong Kong. Previous studies have indicated that the elderly and residents far from the equator have a higher incidence of vitamin D deficiency, and that circulating vitamin D levels are higher in summer [21], [22], [23], [24]. However, the reasons for the lack of association between serum vitamin D level and blood pressure level or risk of hypertension are not known.

The finding that serum PTH level was not associated with blood pressure level or risk of hypertension is in contrast to previous studies. Several studies have consistently demonstrated that higher levels of circulating PTH were associated with higher blood pressure or prevalence of hypertension [14], [17], [4], [13]. In the Longitudinal Ageing Study Amsterdam, higher levels of PTH were significantly associated with higher systolic and diastolic blood pressure and higher prevalence of hypertension [17]. Two recent reports from the US National Health and Nutrition Examination Surveys (NHANES) during 2003–2006 also indicated that serum concentrations of PTH were positively associated with blood pressure [4], [13]. In 7,561 participants over 20 years of age, compared with the lowest PTH quintile (≤ 27 ng/l), adjusted mean difference in blood pressure for the highest quintile (≥59 ng/l) was 5.9 mmHg for systolic blood pressure and 4.5 mmHg for diastolic blood pressure [13]. The lack of association between serum PTH levels and blood pressure, or risk of hypertension might be attributed to the relatively low levels of serum PTH in our study [median concentration 2.83 pmol/l (26.9 ng/l)].

Although we did not find any associations of serum 25(OH)D and PTH levels with blood pressure or risk of hypertension, previous experimental and population studies have provided several mechanisms to explain the relationships among vitamin D, PTH and blood pressure. Vitamin D may influence blood pressure by functioning as an endogenous inhibitor of the renin-angiotensin system (RAS) [25]. And factors modulated by vitamin D include vascular smooth muscle tone [26], vascular endothelium, expression of angiotensin II type 1 receptor, and oxidative stress [27], [28]. PTH has been shown to stimulate renin release by activating the RAS [29]. Additionally, PTH increases blood pressure by direct effects on blood vessels. PTH stimulates vascular smooth muscle cells to produce factors involved in sclerosis [30], and induces the endothelial expression of factors implicated in endothelial dysfunction, such as endothelin-1 and interleukin-6 [31], [32].

A strength of our study is its rapid completion, which minimized seasonal variation of serum 25(OH)D and PTH levels. In addition, there was only 1 participant on calcium supplementation and none received vitamin D supplementation. However, our study has to be interpreted within the context of its limitations. First, we did not collect information on physical activity, dietary vitamin D intake, and sunlight exposure. Second, we used only a single measurement of serum 25(OH)D and PTH. It can be questioned whether serum 25(OH)D and PTH levels measured at a single point in time reflects only recent exposure rather than long-term exposure. In addition, the participants were not selected randomly from the general population. Therefore, the results may not be generalized to the general population. Finally, our study was cross-sectional, and hence no causal inference could be taken.

In conclusion, our study suggests that serum vitamin D and PTH levels are not independently associated with blood pressure or risk of hypertension in a Chinese population. Prospective population studies are needed to evaluate the role of vitamin D and PTH in the incidence of hypertension.

## References

[1] DeLuca HF (1979) The vitamin D system in the regulation of calcium and phosphorus metabolism. Nutr Rev 37: 161–193. 542234 10.1111/j.1753-4887.1979.tb06660.x

[2] Forman JP, Giovannucci E, Holmes MD, Bischoff-Ferrari HA, Tworoger SS, et al (2007) Plasma 25-hydroxyvitamin D levels and risk of incident hypertension. Hypertension 49: 1063–1069. 17372031 10.1161/HYPERTENSIONAHA.107.087288

[3] Kim MK, Il KM, Won OK, Kwon HS, Lee JH, et al (2010) The association of serum vitamin D level with presence of metabolic syndrome and hypertension in middle-aged Korean subjects. Clin Endocrinol (Oxf) 73: 330–338. 20184597 10.1111/j.1365-2265.2010.03798.x

[4] Zhao G, Ford ES, Li C, Kris-Etherton PM, Etherton TD, et al (2010) Independent associations of serum concentrations of 25-hydroxyvitamin D and parathyroid hormone with blood pressure among US adults. J Hypertens 28: 1821–1828. 20613627 10.1097/HJH.0b013e32833bc5b4

[5] Forman JP, Curhan GC, Taylor EN (2008) Plasma 25-hydroxyvitamin D levels and risk of incident hypertension among young women. Hypertension 52: 828–832. 18838623 10.1161/HYPERTENSIONAHA.108.117630 PMC2747298

[6] Jorde R, Figenschau Y, Emaus N, Hutchinson M, Grimnes G (2010) Serum 25-hydroxyvitamin D levels are strongly related to systolic blood pressure but do not predict future hypertension. Hypertension 55: 792–798. 20065152 10.1161/HYPERTENSIONAHA.109.143990

[7] Bhandari SK, Pashayan S, Liu IL, Rasgon SA, Kujubu DA, et al (2011) 25-hydroxyvitamin D levels and hypertension rates. J Clin Hypertens (Greenwich) 13: 170–177. 21366848 10.1111/j.1751-7176.2010.00408.x PMC8672974

[8] Lu L, Yu Z, Pan A, Hu FB, Franco OH, et al (2009) Plasma 25-hydroxyvitamin D concentration and metabolic syndrome among middle-aged and elderly Chinese individuals. Diabetes Care 32: 1278–1283. 19366976 10.2337/dc09-0209 PMC2699709

[9] Chan R, Chan D, Woo J, Ohlsson C, Mellstrom D, et al (2012) Serum 25-hydroxyvitamin D and parathyroid hormone levels in relation to blood pressure in a cross-sectional study in older Chinese men. J Hum Hypertens 26: 20–27. 21248778 10.1038/jhh.2010.126

[10] Dorjgochoo T, Ou SX, Xiang YB, Yang G, Cai Q, et al. (2012) Circulating 25-hydroxyvitamin D levels in relation to blood pressure parameters and hypertension in the Shanghai Women’s and Men’s Health Studies. Br J Nutr: 1–10. 10.1017/S0007114511005745 PMC3806643 22365135

[11] Hagenau T, Vest R, Gissel TN, Poulsen CS, Erlandsen M, et al (2009) Global vitamin D levels in relation to age, gender, skin pigmentation and latitude: an ecologic meta-regression analysis. Osteoporos Int 20: 133–140. 18458986 10.1007/s00198-008-0626-y

[12] Jorde R, Figenschau Y, Emaus N, Hutchinson M, Grimnes G (2010) Serum 25-hydroxyvitamin D levels are strongly related to systolic blood pressure but do not predict future hypertension. Hypertension 55: 792–798. 20065152 10.1161/HYPERTENSIONAHA.109.143990

[13] He JL, Scragg RK (2011) Vitamin D, parathyroid hormone, and blood pressure in the National Health and Nutrition Examination Surveys. Am J Hypertens 24: 911–917. 21525968 10.1038/ajh.2011.73

[14] Taylor EN, Curhan GC, Forman JP (2008) Parathyroid hormone and the risk of incident hypertension. J Hypertens 26: 1390–1394. 18551015 10.1097/HJH.0b013e3282ffb43b

[15] Gu D, Reynolds K, Wu X, Chen J, Duan X, et al (2002) Prevalence, awareness, treatment, and control of hypertension in China. Hypertension 40: 920–927. 12468580 10.1161/01.hyp.0000040263.94619.d5

[16] Levey AS, Coresh J, Balk E, Kausz AT, Levin A, et al (2003) National Kidney Foundation practice guidelines for chronic kidney disease: evaluation, classification, and stratification. Ann Intern Med 139: 137–147. 12859163 10.7326/0003-4819-139-2-200307150-00013

[17] Snijder MB, Lips P, Seidell JC, Visser M, Deeg DJ, et al (2007) Vitamin D status and parathyroid hormone levels in relation to blood pressure: a population-based study in older men and women. J Intern Med 261: 558–565. 17547711 10.1111/j.1365-2796.2007.01778.x

[18] Reis JP, von Mühlen D, Kritz-Silverstein D, Wingard DL, Barrett-Connor E (2007) Vitamin D, parathyroid hormone levels, and the prevalence of metabolic syndrome in community-dwelling older adults. Diabetes Care 30: 1549–1555. 17351276 10.2337/dc06-2438

[19] Margolis KL, Martin LW, Ray RM, Kerby TJ, Allison MA, et al (2012) A prospective study of serum 25-hydroxyvitamin D levels, blood pressure, and incident hypertension in postmenopausal women. Am J Epidemiol 175: 22–32. 22127681 10.1093/aje/kwr274 PMC3291161

[20] Elamin MB, Abu ENO, Elamin KB, Fatourechi MM, Alkatib AA, et al (2011) Vitamin D and cardiovascular outcomes: a systematic review and meta-analysis. J Clin Endocrinol Metab 96: 1931–1942. 21677037 10.1210/jc.2011-0398

[21] Looker AC, Dawson-Hughes B, Calvo MS, Gunter EW, Sahyoun NR (2002) Serum 25-hydroxyvitamin D status of adolescents and adults in two seasonal subpopulations from NHANES III. Bone 30: 771–777. 11996918 10.1016/s8756-3282(02)00692-0

[22] Holick MF (2004) Vitamin D: importance in the prevention of cancers, type 1 diabetes, heart disease, and osteoporosis. Am J Clin Nutr 79: 362–371. 14985208 10.1093/ajcn/79.3.362

[23] Holick MF (2006) High prevalence of vitamin D inadequacy and implications for health. Mayo Clin Proc 81: 353–373. 16529140 10.4065/81.3.353

[24] Rostand SG (1997) Ultraviolet light may contribute to geographic and racial blood pressure differences. Hypertension 30: 150–156. 9260973 10.1161/01.hyp.30.2.150

[25] Li YC, Qiao G, Uskokovic M, Xiang W, Zheng W, et al (2004) Vitamin D: a negative endocrine regulator of the renin-angiotensin system and blood pressure. J Steroid Biochem Mol Biol 89–90: 387–392. 10.1016/j.jsbmb.2004.03.004 15225806

[26] Vaidya A, Forman JP (2010) Vitamin D and hypertension: current evidence and future directions. Hypertension 56: 774–779. 20937970 10.1161/HYPERTENSIONAHA.109.140160

[27] Jablonski KL, Chonchol M, Pierce GL, Walker AE, Seals DR (2011) 25-Hydroxyvitamin D deficiency is associated with inflammation-linked vascular endothelial dysfunction in middle-aged and older adults. Hypertension 57: 63–69. 21115878 10.1161/HYPERTENSIONAHA.110.160929 PMC3020150

[28] Dong J, Wong SL, Lau CW, Lee HK, Ng CF, et al. (2012) Calcitriol protects renovascular function in hypertension by down-regulating angiotensin II type 1 receptors and reducing oxidative stress. Eur Heart J [Epub ahead of print]. 10.1093/eurheartj/ehr459 22267242

[29] Pilz S, Tomaschitz A, Ritz E, Pieber TR (2009) Vitamin D status and arterial hypertension: a systematic review. Nat Rev Cardiol 6: 621–630. 19687790 10.1038/nrcardio.2009.135

[30] Perkovic V, Hewitson TD, Kelynack KJ, Martic M, Tait MG, et al (2003) Parathyroid hormone has a prosclerotic effect on vascular smooth muscle cells. Kidney Blood Press Res 26: 27–33. 12697974 10.1159/000069761

[31] Rashid G, Bernheim J, Green J, Benchetrit S (2007) Parathyroid hormone stimulates endothelial expression of atherosclerotic parameters through protein kinase pathways. Am J Physiol Renal Physiol 292: F1215–1218. 17190908 10.1152/ajprenal.00406.2006

[32] Rubanyi GM, Polokoff MA (1994) Endothelins: molecular biology, biochemistry, pharmacology, physiology, and pathophysiology. Pharmacol Rev 46: 325–415. 7831383

